# Effect of Suction/Injection on Unsteady Hydromagnetic Convective Flow of Reactive Viscous Fluid between Vertical Porous Plates with Thermal Diffusion

**DOI:** 10.1155/2014/980270

**Published:** 2014-11-04

**Authors:** I. J. Uwanta, M. M. Hamza

**Affiliations:** Department of Mathematics, Usmanu Danfodiyo University, PMB 2346, Sokoto, Nigeria

## Abstract

An investigation is performed to study the effect of suction/injection on unsteady hydromagnetic natural convection flow of viscous reactive fluid between two vertical porous plates in the presence of thermal diffusion. The partial differential equations governing the flow have been solved numerically using semi-implicit finite-difference scheme. For steady case, analytical solutions have been derived using perturbation series method. Suction/injection is used to control the fluid flow in the channel, and an exothermic chemical reaction of Arrhenius kinetic is considered. Numerical results are presented graphically and discussed quantitatively with respect to various parameters embedded in the problem.

## 1. Introduction

Suction or injection on the boundary layer control played significant role in the field of aerodynamics and space sciences. Shojaefard et al. [1] used suction/injection to control fluid flow on the surface of subsonic aircraft. By controlling the flow as such, fuel consumption might be decreased by 30%, a considerable reduction in pollutant emission is achieved, and operating costs of commercial airplanes are reduced by at least 8%; see the study by Braslow in [2]. In mass transfer cooling, suction or injection of a fluid through the bounding surface can significantly change the flow field and, as a result, affect the heat transfer rate from the plate; see the study by Ishak et al. in [3]. Many interests have been built in the study of flow of heat and mass transfer with suction or injection because of its extensive engineering applications. In the area of steady flow of viscous incompressible fluid over infinite porous plates subject to suction or injection, various aspects of the problem have been investigated by many authors. To be more specific, Griffith and Meredith [4] investigated the steady flow of an incompressible viscous fluid over an infinite porous flat plate subject to uniform suction. Jena and Mathur [5] studied free convection in the laminar boundary layer flow of a thermomicropolar fluid over a vertical flat plate subject to uniform suction or injection. Boundary layer controls by suction or injection in the flow of incompressible fluid over an infinite porous wedge are to be found in the studies by Devi and Kandasamy [6] and Kandasamy et al. [7, 8]. Layek et al. [9], Shateyi [10], and Cortell [11] have analyzed the stretching sheet problem with suction or injection. Attia [12] reported the unsteady flow due to a rotating disk with uniform suction or injection. Al-Sanea [13] investigated mixed convection heat transfer along a continuously moving heated vertical plate with suction or injection. Unsteady free convection and mass transfer flow over an infinite vertical porous plate considering suction or injection are to be found in the study by Takhar et al. in [14]. Recently, Cortell [15] studied the effects of suction, viscous dissipation, and thermal radiation on flow and heat transfer of a power-law fluid past an infinite porous plate. Effect of suction and injection on unsteady free convection Couette flow and heat transfer of reactive viscous fluid in vertical porous plate is to be found in the study by Jha et al. in [16].

The investigation of the flow of an electrically conducting fluid in a porous channel in the presence of a transverse magnetic field is important because of its widespread engineering and industrial applications such as MHD marine propulsion, electronic packages, microelectronic devices, thermal insulation, petroleum reservoirs, MHD stirring of molten metal, exothermic reaction in packaged reactors, and magnetic-levitation casting. On the other hand, Soret or thermal diffusion is important where more than one chemical species are present under very large temperature gradients, such as chemical reactions and isotope separation, and in mixtures of gases with very light molecular weight such as hydrogen or helium and of medium molecular weight such as nitrogen or air. Because of the applications of MHD and Soret, many authors investigated their effects on natural convection heat and mass transfer flow. Postelnicu [17] reported the influence of magnetic field on heat and mass transfer by natural convection from vertical surfaces in porous media considering Soret and Dufour effects. Osalusi et al. [18] determined numerically the effects of Soret and Dufour on heat and mass transfer of a steady MHD convective and slip flow due to a rotating disk with viscous dissipation and ohmic heating. Recently, Turkyilmazoglu and Pop [19] reported the effects of Soret and heat source on unsteady radiative MHD free convection flow from an impulsively infinite vertical plate. In nutshell, there have been considerable published works dealing with steady flow with Soret and Dufour effect; some of them are the works of Alam et al. [20], Chamkha and Ben-Nakhi [21], Tsai and Huang [22], Tak et al. [23], and Magyari and Postelnicu [24]. Steady flows with chemical reaction considering Soret and Dufour effect are to be found in the studies by Mansour et al. [25], Beg et al. [26], El-Kabeir et al. [27], and Gangadhar [28]. Unsteady fluid flow problems in the presence of Soret and Dufour effects with chemical reaction can be found in the studies by Bhargava et al. [29] and Pal and Mondal [30]. Nandkeolyar et al. [31] investigated numerically and analytically the effect of suction/injection on unsteady hydromagnetic heat and mass transfer flow of a radiating and chemically reactive fluid past a flat porous plate with ramped wall temperature. Numerical investigation of buoyancy effects on hydromagnetic unsteady flow through a porous channel considering suction and injection is to be found in the study by Makinde and Chinyoka [32].

The aim of the present analysis is to study the effect of suction/injection on unsteady hydromagnetic convective flow of viscous reactive fluid between two infinite vertical parallel porous plates in the presence of transverse magnetic field and thermal diffusion. In this paper, an exothermic chemical reaction of Arrhenius kinetics is employed and suction/injection is used to control fluid flow in the channel.

## 2. Governing Equations

**Figure 1:**
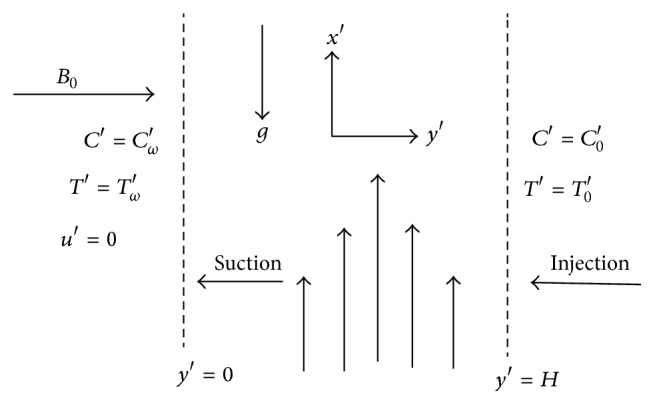
Schematic diagram of the problem.

Consider the transient natural convection and mass transfer flow of viscous reactive, incompressible, and electrically conducting fluid between infinite vertical parallel porous plates under the influence of a transversely magnetic field of strength *B*_0_; see Figure 1. The magnetic Reynolds number is assumed to be small so that the induced magnetic field and the hall effect of MHD are negligible. At time *t*′ ≤ 0, both the fluid and the plates are at rest and at the same temperature and concentration *T*_0_′ and *C*_0_′, respectively. At *t*′ > 0, the temperature and concentration of the plate *y*′ = 0 are raised to *T*_*ω*_′ and *C*_*ω*_′ and thereafter remain constant and those of *y*′ = *H* are lowered to *T*_0_′ and *C*_0_′, where *T*_*ω*_′ > *T*_0_′ and *C*_*ω*_′ > *C*_0_′. It is assumed that the flow is subjected to suction of the fluid from one porous plate and at the same rate fluid is being injected through the other porous plate. We chose a Cartesian coordinate system with the *x*′ axis along the upward direction and the *y*′ axis normal to it. The physical properties are assumed to be constant excluding density in the buoyancy term. The fluid is assumed to be Newtonian and obeys the Boussinesq's approximation. Under the previous assumptions, the momentum, energy, and concentration equations in the dimensional form are the following:

(1) ∂u′∂t′−v0∂u′∂y′=ν∂2u′∂y′2∂u′∂t′−v0∂u′∂y′=+gβ(T′−T0′)+gβ∗(T′−T0′)−σB02ρu′,∂T′∂t′−v0∂T′∂y′=kρCp∂2T′∂y′2+QC0AρCpe−E/RT′,∂C′∂t′−v0∂C′∂y′=Dm∂2C′∂y′2+DmkTTm∂2T′∂y′2.

The initial and boundary conditions for the present problem are the following:

(2) $$\begin{array}{c} t^{\prime }\leq 0:u^{\prime }=0,\;T^{\prime }⟶T_{0}^{\prime },\;C^{\prime }⟶C_{0}^{\prime },\;0\leq y^{\prime }\leq H,\\ t^{\prime }>0:u^{\prime }=0,\;T^{\prime }=T_{\omega }^{\prime },\;C^{\prime }=C_{\omega }^{\prime }\;\text{at}\;y^{\prime }=0,\\ u^{\prime }=0,\;T^{\prime }=T_{0}^{\prime },\;C^{\prime }=C_{0}^{\prime }\;\text{as}\;y^{\prime }⟶H, \end{array}$$

where *σ* is the conductivity of the fluid, *B*_0_ is the electromagnetic induction, *β* is the coefficient of thermal expansion, *β* ^*^ is the coefficient of concentration expansion, *Q* is the heat of reaction, *A* is the rate constant, *E* is the activation energy, *R* is the universal gas constant, *ν* is the kinematic viscosity, *C*_0_′ is the initial concentration of the reactant species, *g* is the gravitational force, *C*_*p*_ is the specific heat at constant pressure, *k* is the thermal conductivity of the fluid, *ρ* is the density of the fluid, *D*_*m*_ is the coefficient of mass diffusivity, *T*_*m*_ is the mean fluid temperature, and *k*_*T*_ is the thermal diffusion ratio.

In order to solve (1) to (2), we employ the following dimensionless parameters:

(3) y=y′H,    t=t′νH2,  u=u′ν0,Pr⁡=νρCpk,  Gc=gβ∗RC02H2Eνν0,  Gr=gβRT02H2Eνν0,θ=ET′−T0RT02,    λ=QC0AEH2RT02e−E/RT0,Sc=νDm,  Sr=kTT02TmC02,  C=EC′−C0RC02,ε=RT0E,  θT=E(Tω−T0)RT02,  CT=E(Cω−C0)RC02,γ=ν0Hν,    M=σB02H2νρ.

Using (3), (1) to (2) can take the following form:

(4) $$\begin{array}{c} \frac{\partial u}{\partial t}-\gamma \frac{\partial u}{\partial y}=\frac{\partial^{2}u}{\partial y^{2}}+\text{Gr}\theta +\text{Gc}C-Mu,\\ \mathrm{Pr}\left(\frac{\partial \theta }{\partial t}-\gamma \frac{\partial \theta }{\partial y}\right)=\frac{\partial^{2}\theta }{\partial y^{2}}+\lambda e^{\left(\theta /\left(1+\varepsilon \theta \right)\right)},\\ \text{Sc}\left(\frac{\partial C}{\partial t}-\gamma \frac{\partial C}{\partial y}\right)=\frac{\partial^{2}C}{\partial y^{2}}+\text{Sr}\frac{\partial^{2}\theta }{\partial y^{2}}. \end{array}$$

The initial and boundary conditions in dimensionless form are the following:

(5) $$\begin{array}{c} u=0,\;\theta =0,\;0\leq y\leq 1,\;\;\;t\leq 0,\\ t>0:u=0,\;\theta =\theta_{T},\;C=C_{T}\;\text{at}\;y=0,\\ u=0,\;\theta =0,\;C=0,\;\text{as}\;y=1. \end{array}$$

## 3. Analytical Solutions

The analytical solutions have played an important role in validating and exploring computer routines of complicated problems. They are also used to inspect the internal consistency of mathematical models and of the approximations adopted by Jha et al. in [16]. Therefore, we reduce the governing equations of this problem due to its nonlinearity into a form that can be solved analytically. By setting ∂*u*/∂*t* = 0, ∂*θ*/∂*t* = 0, and ∂*C*/∂*t* = 0 into (4) to (5) and by taking *θ*_*T*_ = 1 and *C*_*T*_ = 1 at the boundary, we get

(6) −γ∂u∂y=∂2u∂y2+Grθ+GcC−Mu,−γPr⁡∂θ∂y=∂2θ∂y2+λeθ/1+εθ,−γSc∂C∂y=∂2C∂y2+Sr∂2θ∂y2.

The boundary conditions are the following:

(7) u=0, θ=1, C=1, at  y=0,u=0, θ=0, C=0, at  y=1.

In order to construct an approximate solution to (6) subject to (7), we employed a regular perturbation method by taking a power series expansion in the Frank-Kamenetskii parameter *λ* as follows:

(8) u=u0+λu1,C=C0+λC1,θ=θ0+λθ1.

Substituting (8) into (6), the solutions of the governing equations are obtained as follows:

(9) u=H1eh1y+H2e−h2y+H3+H4e−γPr⁡y+H5e−γScy+λH6eh1y+H7e−h2y+H8y+H9  +H10+H11e−γPr⁡y+H12ye−γPr⁡y  +H13e−2γPr⁡y+H14e−γScy,θ=1+Be−γPr⁡y−1+λD1+D2e−γPr⁡y+D3y  +D4ye−γPr⁡y+D5e−2γPr⁡y,C=E1+E2e−γScy+E3e−γPr⁡y+λE4+E5e−γScy+(E6+E7)e−γPr⁡y  +E8ye−γPr⁡y+E9e−2γPr⁡y.

Using (9), we write the steady-state skin friction, rate of heat transfer, and rate of mass transfer on the boundaries as follows.

Steady-state skin frictions on the boundary plates are the following:

(10) ∂u∂yy=0=H1h1−H2h2−γPr⁡H4−γScH5+λH6h1−H7h2+H8  −H10+H11+2H13+H12−H14Scγ,∂u∂yy=1=H1h1eh1−H2h2e−h2−γPr⁡H4e−γPr⁡−γScH5e−γSc+λH6h1eh1−H7h2e−h2+H8  +H12+H12γPr⁡−H11γPr⁡−H10γPr⁡e−γPr⁡  −2H13γPr⁡e−2γPr⁡−H14Scγe−γSc.

The steady-state rate of heat transfer on the boundary plates is

(11) −∂θ∂yy=0=−−BγPr⁡+λ−D2γPr⁡+D3+D4−2D5γPr⁡, ∂θ∂yy=1=−BγPr⁡e−γPr⁡ ∂θ∂yy=1=+λD3+(D4−D4γPr⁡−D2γPr⁡)e−γPr⁡ ∂θ∂yy=1=+λ00−2γPr⁡e−2γPr⁡.

The steady-state rate of mass transfer on the boundary plates is given as follows:

(12) −∂C∂yy=0 =−−γScE2−γPr⁡E3    +λ−E5γSc−γPr⁡⁡E6+E7+2E9+E8,∂C∂yy=1 =−γScE2e−γSc−γPr⁡E3e−γPr⁡  +λ−γScE5e−γSc    +E8−E8γPr⁡−E6γPr⁡−E7γPr⁡e−γPr⁡    −2γPr⁡E9e−2γPr⁡.

The constants *B*, *H*_1_, *H*_2_, *H*_3_, *H*_4_, *H*_5_, *H*_6_, *H*_7_, *H*_8_, *H*_9_, *H*_10_, *H*_11_, *H*_12_, *H*_13_, *H*_14_, *D*_1_, *D*_2_, *D*_3_, *D*_4_, *D*_5_, *E*_1_, *E*_2_, *E*_3_, *E*_4_, *E*_5_, *E*_6_, *E*_7_, *E*_8_, *E*_9_, *h*_1_, and *h*_2_ are defined in the appendix section.

## 4. Numerical Solutions

**Figure 2:**
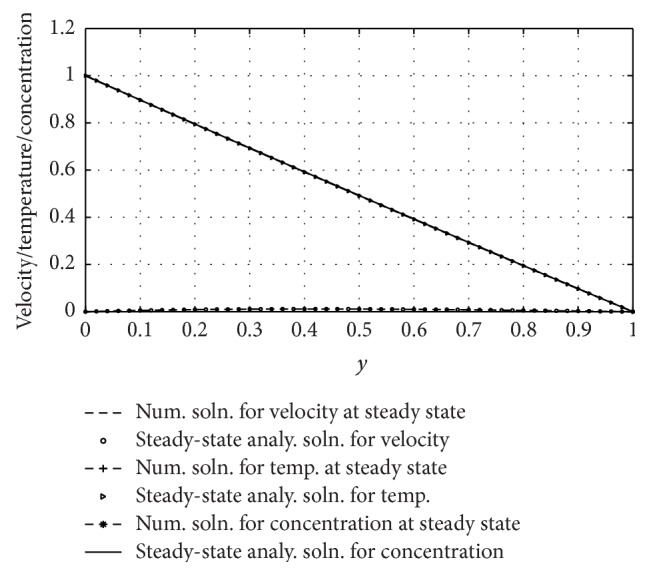
Unsteady and steady-state solutions for velocity, concentration, and temperature profiles.

The complete forms of (4) are solved numerically using semi-implicit finite-difference method given in the study by Makinde and Chinyoka in [32]. We used forward difference formulas for all time derivatives and approximate the spatial derivatives with central difference formula. The semi-implicit finite-difference equations corresponding to (4) are as follows:

(13) −r1uj−1(N+1)+(1+2r1)uj(N+1)−r1uj+1(N+1) =r2uj−1(N)+(1−2r2−γr3−MΔt)uj(N)  +(r2+γr3)uj+1N+ΔtGrθjN+ΔtGcCjN,−r1Cj−1(N+1)+(Sc+2r1)Cj(N+1)−r1Cj+1(N+1) =r2Cj−1(N)+(Sc−2r2−Scγr3)Cj(N)  +r2+Scγr3Cj+1N+r4θj−1N−2θjN+θj+1N,−r1θj−1(N+1)+(Pr⁡+2r1)θj(N+1)−r1θj+1(N+1) =r2θj−1(N)+(Pr⁡−2r2−γPr⁡r3)θj(N)  +r2+γPr⁡r3θj+1N+λΔtexp⁡θjN1+εθjN,

where *r*_1_ = *ξ*Δ*t*/Δ*y* ^2^, *r*_2_ = (1 − *ξ*)Δ*t*/Δ*y* ^2^, *r*_3_ = Δ*t*/Δ*y*, *r*_4_ = SrΔ*t*/Δ*y* ^2^, and 0 ≤ *ξ* ≤ 1. We chose *ξ* = 1; the detailed reasons to this particular selection are documented in [32]. Also the analytical solutions displayed in the previous section are used as a check on the accuracy and effectiveness of the numerical scheme. Again, in order to reconfirm the accuracy of the scheme, the numerical results for velocity, concentration, and temperature are compared with the analytical solutions. It has been found that the numerical values of the velocity, concentration, and temperature fields calculated from the expressions (9) have matched very well with the numerical obtained from the expressions (13) at the steady-state time. See Figure 2 for the graph of the numerical solutions at steady state and steady-state analytical solutions for velocity, concentration, and temperature fields.

## 5. Results and Discussion

The numerical results are obtained by solving (13) using the method described in the previous section for various values of physical parameters to describe the physics of the problem. The nondimensional parameters that govern the flow are the Prandtl number (Pr), which is inversely proportional to the thermal diffusivity of the working fluid, the Frank-Kamenetskii parameter (*λ*), the Soret number (Sr), the magnetic parameter (*M*), the thermal Grashof number (Gr), the solutal Grashof number (Gc), the nondimensional time (*t*), the Schmidt number (Sc), which is inversely proportional to the mass diffusivity of the working fluid, and suction/injection parameter (*γ*), which were simultaneously applied each to opposite porous plates of the channel at the same rate. For the purpose of discussion, some numerical calculations are carried out for dimensionless velocity (*u*), temperature (*θ*), concentration (*C*), skin friction, rate of heat transfer in terms of Nusselt number, and the rate of mass transfer in terms of Sherwood number. Unless otherwise stated, the values *λ* = 0.1, Gr = 0.1, *M* = 1, Gc = 0.1, Sr = 0.1, *θ*_*T*_ = 1, *C*_*T*_ = 1, *Pr*⁡ = 0.71, Sc = 0.62, *t* = 0.1, *γ* = 0.5, and *ε* = 0.01 are used for the investigation. Results obtained are displayed graphically for velocity, temperature, concentration, skin friction, Nusselt number, and Sherwood number for various flow parameters.

**Figure 3:**
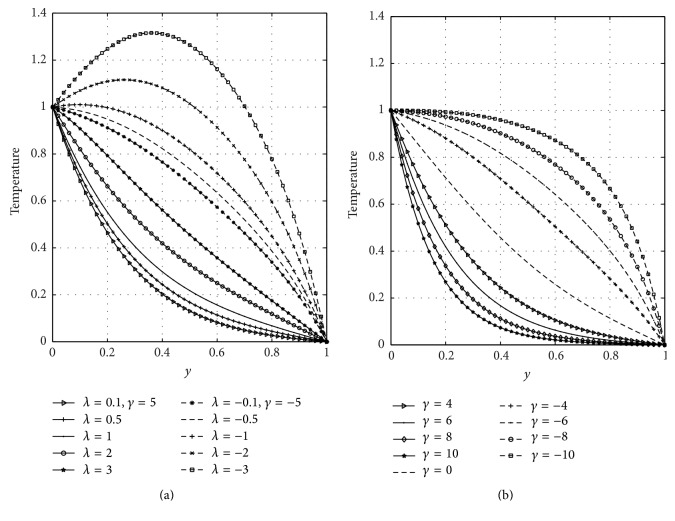
Effects of Frank-Kamenetskii parameter (*λ*) and suction/injection (*γ*) on temperature.

**Figure 4:**
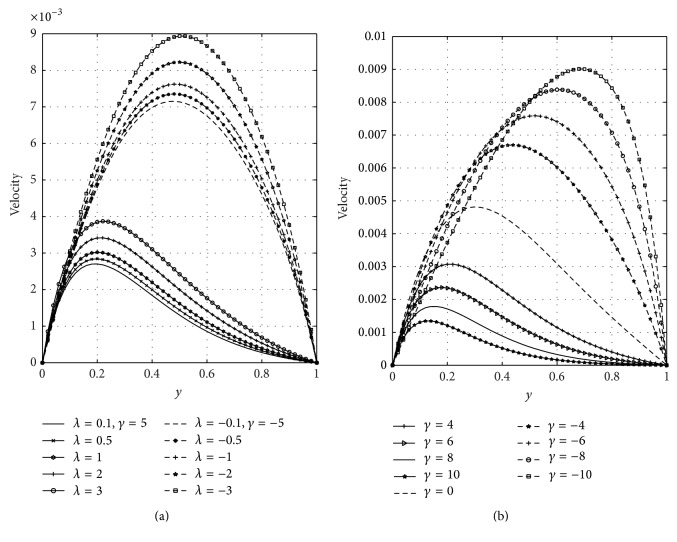
Effects of Frank-Kamenetskii parameter (*λ*) and suction/injection (*γ*) on velocity.

Figure 3 shows the effects of the Frank-Kamenetskii parameter (*λ*) and suction/injection (*γ*) on the temperature profiles. From Figure 3(a), it is observed that temperature of the fluid increases with increasing values of *λ* in case of suction and injection, respectively. This is physically true since an increase in *λ* leads to significant increases in the reaction and viscous source terms and hence significantly increases the fluid temperature. It is evident from Figure 3(a) that temperature of the fluid is greater in case of injection than suction.  Figure 3(b) represents the influence of suction/injection parameter on the temperature field. From Figure 3(b), it is seen that temperature decreases due to suction but increases due to injection. In case of suction, the fluid at ambient conditions is brought closer to the surface and reduces the thermal boundary layer thickness. The same principle operates but in reverse direction in case of injection. Figures 4(a) and 4(b) illustrated the effects of the Frank-Kamenetskii parameter (*λ*) and suction/injection parameter (*γ*) on velocity distribution, respectively.  Figure 4(a) revealed that increasing *λ* accelerates the velocity of the fluid in case of suction and injection. It is evident from this figure that the velocity is higher in case of injection than suction. From Figure 4(b), it is seen that velocity of the fluid decelerates due to suction while it accelerates due to blowing. The physical explanation for such a behavior is that while stronger blowing is provided, the heated fluid is pushed farther from the wall where the buoyancy forces can act to accelerate the flow with less influence of the viscosity. This effect acts to increase the shear by increasing the maximum velocity within the boundary layer. The same principle operates but in reverse direction in case of suction. It is also noticed that, in case of suction, velocity of the fluid moves away from the channel centerline towards the plate (*y* = 0) and, in case of injection, the maximum velocities are shifted towards the right porous plate (*y* = 1).

**Figure 5:**
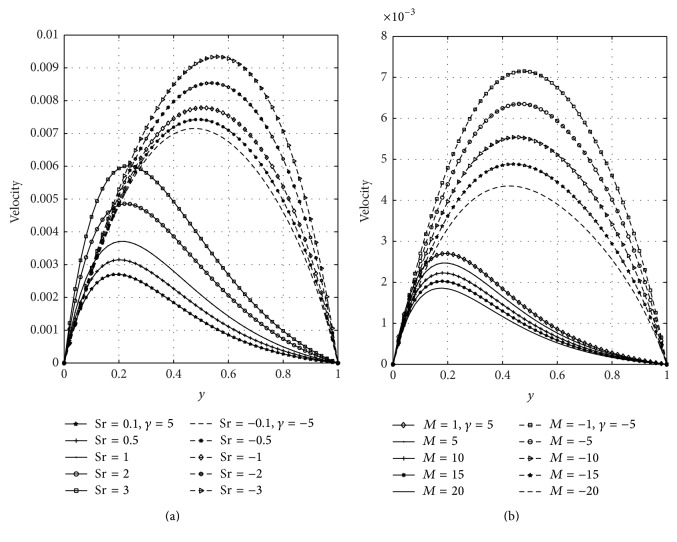
Effects of Soret number (Sr) and magnetic parameter (*M*) on velocity.

**Figure 6:**
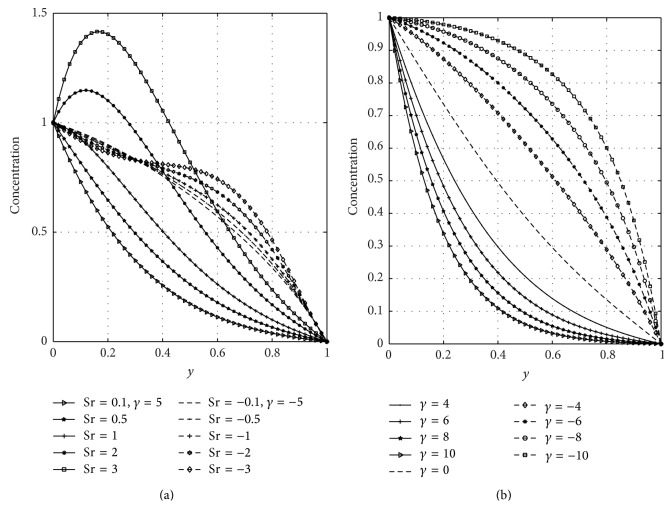
Effects of Soret number (Sr) and suction/injection (*γ*) on concentration.

**Figure 7:**
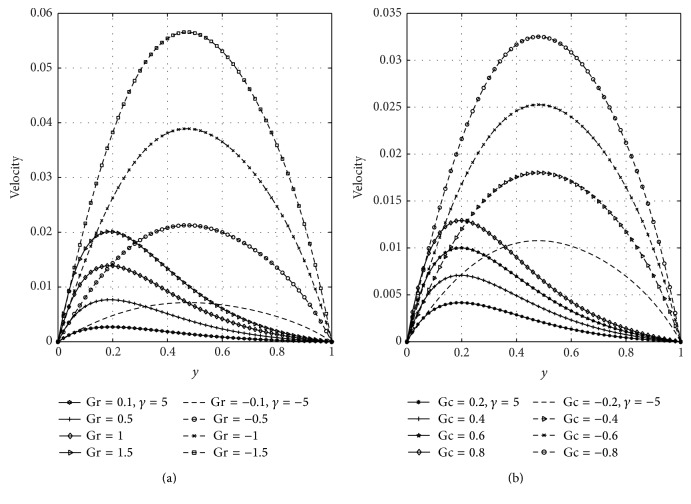
Effects of thermal Grashof number (Gr) and solutal Grashof number (Gc) on velocity.

In Figure 5, we have presented the response of the fluid velocity to variations in the Soret number (Sr) and magnetic parameter (*M*) in the presence of suction and injection parameter.  Figure 5(a) shows the effect of the Soret number on the velocity with constant suction and injection. From this figure, it is noted that Soret number accelerates the fluid velocity in the presence of suction and injection. In Figure 5(a), it is seen that, in the presence of suction, the velocity of the fluid moves toward the left porous plate and in case of injection the maximum velocities move towards the right porous plate.  Figure 5(b) revealed that increasing the strength of magnetic parameter is to decrease velocity profiles. This is due to the fact that transverse magnetic field produces a resistivity force (Lorentz force) similar to the drag force which retards the velocity. It is seen that the velocity of the fluid is greater in case of injection than suction. The effects of the Soret number (Sr) and suction/injection parameter (*γ*) on the concentration distribution are shown graphically in Figures 6(a) and 6(b), respectively. From Figure 6(a), it can be noticed that concentration of the fluid increases with increasing values of Sr in case of suction and injection. Further, Figure 6(a) reveals that the concentration of the fluid is high in the vicinity of the wall (*y* = 0), where suction takes place more than at the wall (*y* = 1) where injection takes place. From Figure 6(b), it can be noticed that concentration of the fluid decreases due to suction but increases due to injection. The influence of the thermal Grashof number (Gr) and solutal Grashof number (Gc) is illustrated in Figures 7(a) and 7(b), respectively. These plots of Figures 7(a) and 7(b) indicate that the momentum boundary layer thickness increases with increasing values of Gr and Gc. It is further noticed from these figures that velocity of the fluid is greater in case of injection than suction.

**Figure 8:**
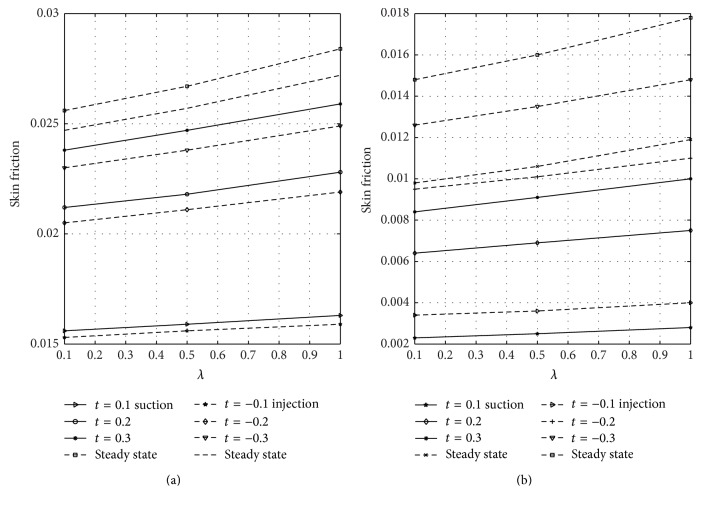
Variation of skin friction with *λ* and time (*t*) at *y* = 0 and *y* = 1.

**Figure 9:**
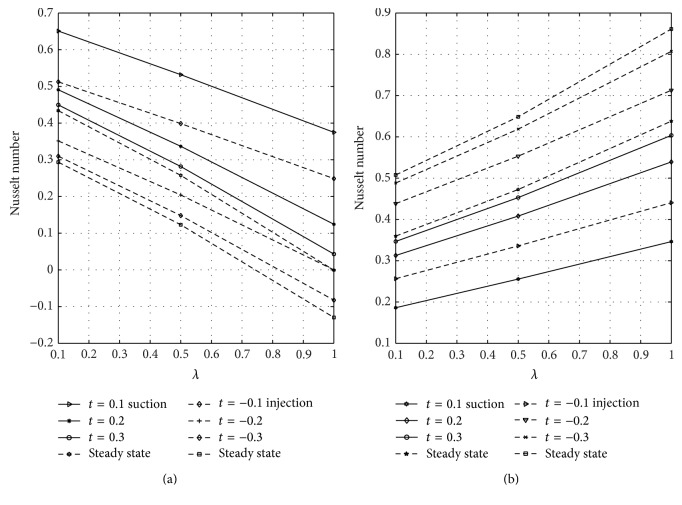
Variation of Nusselt number with *λ* and time (*t*) at *y* = 0 and *y* = 1.

**Figure 10:**
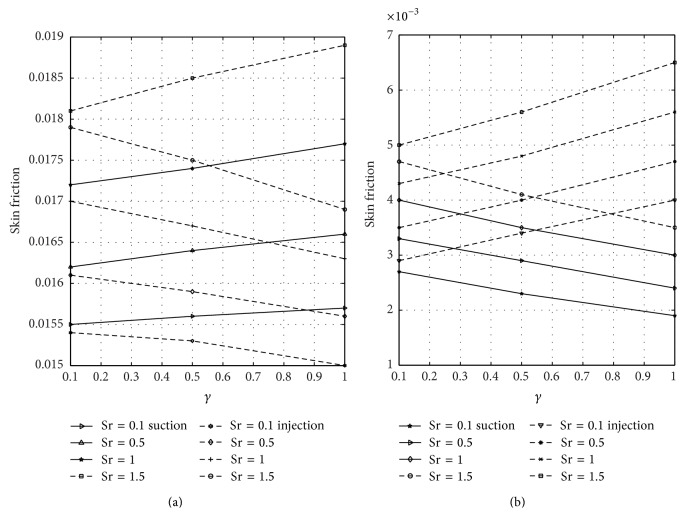
Variation of skin friction with Sr and suction/injection (*γ*) at *y* = 0 and *y* = 1.

**Figure 11:**
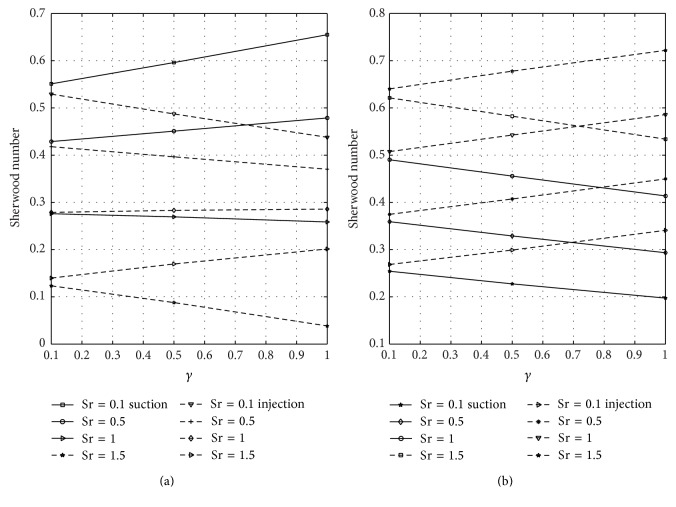
Variation of Sherwood number with Sr and suction/injection (*γ*) at *y* = 0 and *y* = 1.

The wall shear stress dependence on reaction parameter *λ* is illustrated in Figure 8 for varying values of the nondimensional time when suction and injection take place. Figures 8(a) and 8(b) represent the wall shear stress at the walls *y* = 0 and *y* = 1, respectively. From Figure 8, it is observed that skin friction increases as time and *λ* increase until a steady-state condition is reached in case of suction and injection.  Figure 8(a) reveals that skin friction is greater in case of suction than injection, but opposite trend is noticed in Figure 8(b). The rate of heat transfer in terms of Nusselt number dependence on the reaction parameter *λ* is displayed in Figure 9 for varying values of time in the presence of suction and injection. Figures 9(a) and 9(b) represent the Nusselt number at *y* = 0 and *y* = 1 for different values of *λ*, respectively. From Figure 9(a), it is seen that the rate of heat transfer decreases by increasing time and *λ* until a steady-state condition is attained in case of suction and injection. It is also evident from Figure 9(a) that Nusselt number decreases more in case of injection than suction.  Figure 9(b) reveals that rate of heat transfer at the plate *y* = 1 increases as time and *λ* increase until a steady-state value is achieved. From this figure, it is seen that Nusselt number is higher in case of injection than suction. The skin friction on suction/injection parameter for varying values of the Soret number is illustrated in Figure 10. Figures 10(a) and 10(b) represent the skin friction at the walls *y* = 0 and *y* = 1, respectively. From Figure 10(a), it is noticed that skin friction increases as Sr increases in case of suction while it decreases due to injection.  Figure 10(b) reveals that skin friction increases with increasing values of Sr in case of injection while it decreases due to suction.  Figure 11 shows the influence of Sr on the Sherwood number. Figures 11(a) and 11(b) represent the Sherwood number at the plates *y* = 0 and *y* = 1. Sherwood number decreases as Sr increases in case of suction and injection; see Figure 11(a).  Figure 11(b) reveals that Sherwood number increases as Sr increases in case of injection while it decreases as Sr increases due to suction.

**Figure 12:**
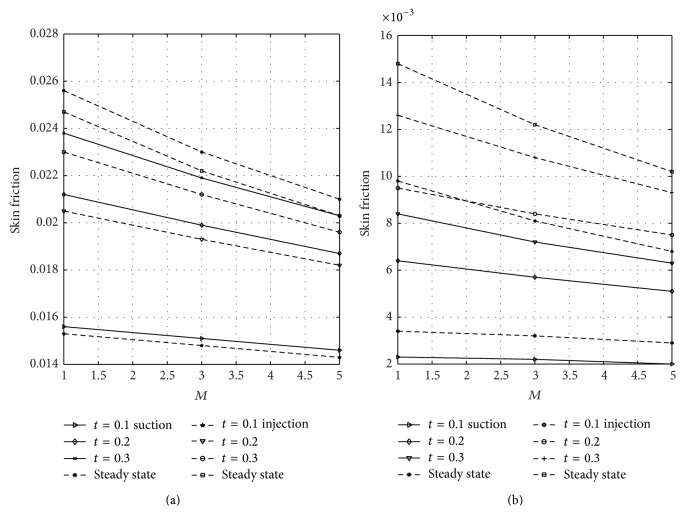
Variation of skin friction with magnetic parameter (*M*) at *y* = 0 and *y* = 1.

**Figure 13:**
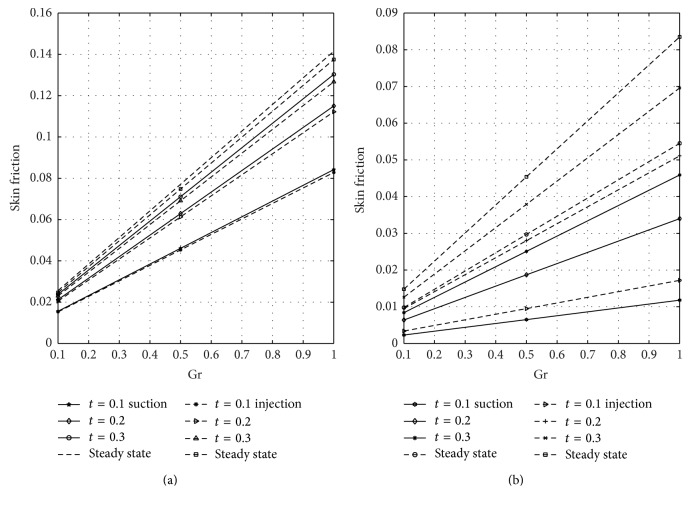
Variation of skin friction with thermal Grashof number (Gr) at *y* = 0 and *y* = 1.

**Figure 14:**
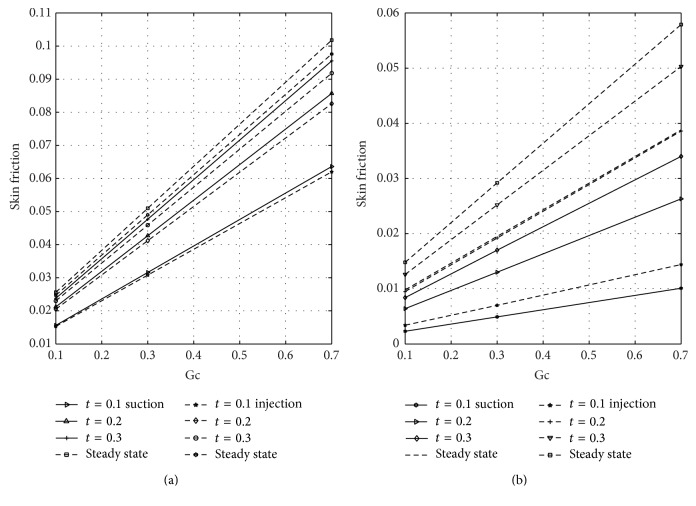
Variation of skin friction with solutal Grashof number (Gc) at *y* = 0 and *y* = 1.

Figure 12 shows the wall shear stress dependence on magnetic parameter *M* at the plates *y* = 0 and *y* = 1 for varying values of nondimensional time in case of suction and injection. Figures 12(a) and 12(b) represent the skin friction at the plates *y* = 0 and *y* = 1, respectively. Figure 12 reflected that skin friction increases with increasing time until a steady-state condition is attained in case of suction and injection.  Figure 12(a) revealed that higher values of *M* reduce the skin friction in case of suction and injection. Reduction is noticed in case of injection more than suction. From Figure 12(b), it is seen that as *M* increases the skin friction decreases in the presence of suction and injection. Further, it is observed from Figure 12(b) that reduction is seen in the presence of suction more than injection when *M* is large. The wall shear stress dependence on Gr for varying values of time at the porous plates *y* = 0 and *y* = 1 when suction and injection take place is illustrated in Figure 13. Figures 13(a) and 13(b) represent the skin friction at the walls *y* = 0 and *y* = 1, respectively.  Figure 14 shows the wall shear stress dependence on Gc for varying values of time when suction and injection take place. Figures 14(a) and 14(b) represent the skin friction at the porous plates *y* = 0 and *y* = 1, respectively. Figures 13 and 14 revealed that skin friction increases by increasing nondimensional time until a steady-state value is achieved. In both Figures 13(a) and 14(a), skin friction when suction takes place is greater than when injection takes place, but reverse effect is observed in Figures 13(b) and 14(b).

## 6. Conclusion

In the present study, the effect of suction/injection on transient hydromagnetic convective flow of viscous reactive fluid between vertical porous plates in the presence of transverse magnetic field and thermal diffusion is investigated. It is found that suction/injection, thermal diffusion, reaction consumption, and thermal and solutal buoyancy play an important role in controlling the transport phenomena. Formation of the minimum flow occurs near the wall where suction takes place, while the maximum flow forms near the wall where injection takes place except in the concentration distribution for varying values of thermal diffusion. It is hoped that the present work may be useful in engineering applications where the formation of boundary layer is to be delayed or enhanced.

## Appendix

Consider the following:

(A.1) Bj=−1e−γPr⁡−1,  a=ε−12,D3=−2−B−a1−B2γPr⁡,  D4=−21−BBa−BγPr⁡,D5=B2aγ2Pr2,  D1=−D2−D5,D2=−D5e−2γPr⁡−1e−γPr⁡−1−D4e−γPr⁡e−γPr⁡−1−D3e−γPr⁡−1,E1=1−E2−E3,  E2=−1e−γSc−1−E3e−2γPr⁡−1e−γSc−1,E3=−Srγ2Pr2Bγ2Pr2−Scγ2Pr⁡,  E6=−SrD2γ2Pr2−2γPr⁡D4γ2Pr2−Scγ2Pr⁡,E8=−SrD4γ2Pr2γ2Pr2−Scγ2Pr⁡,  E7=−E8γSc−2γPr⁡γ2Pr2−Scγ2Pr⁡,E9=−4SrD5γ2Pr24γ2Pr2−2Scγ2Pr⁡,  E4=−E5−E6−E7−E9,h1=−γ+γ2+4M2,  h2=γ+γ2+4M2,H3=Gr1−B+GcE1M,  H4=−GrB+GcE3γ2Pr2−γ2Pr⁡−M,H5=−GcE2γ2Sc2−γ2Sc−M,  H1=−H2−H3−H4−H5,H2=H3eh1−1e−h2−eh1+H4eh1−e−γPr⁡e−h2−eh1+H5eh1−e−γSce−h2−eh1,H8=GrD3M,  H9=GrD1+GcE4+γH8M,H10=−GrD2+GcE6+GcE7γ2Pr2−γ2Pr⁡−M,H11=H12γ+2γPr⁡γ2Pr2−γ2Pr⁡−M,H12=−GrD4+GcE8γ2Pr2−γ2Pr⁡−M,  H13=−GrD5+GcE94γ2Pr2−2γ2Pr⁡−M,H14=−GcE5γ2Sc2−γ2Sc−M,H6=−H7−H9−H10−H11−H13−H14,H7=−H8e−h2−eh1+H9eh1−1e−h2−eh1  +H10eh1−e−γPr⁡e−h2−eh1+H11eh1−e−γPr⁡e−h2−eh1  −H12e−γPr⁡e−h2−eh1  +H13eh1−e−2γPr⁡e−h2−eh1+H14eh1−e−γSce−h2−eh1.
